# Comparative effectiveness of surgery versus external beam radiation with/without brachytherapy in high‐risk localized prostate cancer

**DOI:** 10.1002/cam4.2605

**Published:** 2019-11-07

**Authors:** Ming Yin, Jing Zhao, Paul Monk, Douglas Martin, Edmund Folefac, Monika Joshi, Ning Jin, Amir Mortazavi, Claire Verschraegen, Steven Clinton

**Affiliations:** ^1^ Division of Medical Oncology The Ohio State University Comprehensive Cancer Center Columbus OH USA; ^2^ Biomedical Statistics The Ohio State University Comprehensive Cancer Center Columbus OH USA; ^3^ Radiation Oncology The Ohio State University Comprehensive Cancer Center Columbus OH USA; ^4^ Division of Hematology and Oncology Penn State University Hershey Cancer Institute Hershey PA USA

**Keywords:** prostate cancer, radiation therapy, surgery, survival

## Abstract

**Background:**

It remains controversial if radical prostatectomy or definitive radiation therapy produces equivalent outcomes in high‐risk localized prostate cancer.

**Methods:**

We queried The Surveillance, Epidemiology, and End Results (SEER) database for those who received upfront surgery or who were recommended for surgery but instead received radiation. Inverse probability of treatment weighing was used to adjust for covariate imbalance and the weighted Cox proportional hazards model was used to estimate the effects of treatment groups on survival. A meta‐analysis was performed to pool estimates from published studies.

**Results:**

Among eligible 62 533 patients, 59 540 had upfront surgery and 2993 patients had upfront radiotherapy. EBRT + BT was associated with a superior cancer‐specific survival (CSS) compared with surgery or EBRT alone (HR, 0.55, 95% CI, 0.3‐1.0; HR, 0.49, 95% CI, 0.24‐0.98, respectively), whereas EBRT was associated with an inferior overall survival (OS) compared with surgery (HR, 1.46, 95% CI, 1.16‐1.8). Radiotherapy (EBRT ± BT) was inferior to surgery by OS (HR, 1.63, 95% CI, 1.13‐2.34) in patients ≤ 65 years, and was superior to surgery by CSS in patients > 65 years (HR, 0.69, 95% CI, 0.49‐0.97). The meta‐analysis showed consistent results.

**Conclusion:**

EBRT + BT was associated with a significantly better prostate CSS compared with surgery or EBRT. EBRT alone was inferior to surgery by OS.

## Graphical abstract

In high‐risk localized prostate cancer, radiotherapy with brachytherapy boost was associated with a longer survival outcome, compared with surgery or radiotherapy without brachytherapy boost.

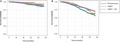

## 1 INTRODUCTION

High‐risk prostate cancer, characterized by prostate‐specific antigen (PSA) > 20 ng/mL, Gleason score ≥ 8, or clinical T stage ≥ 3, accounts for approximately 15% of all prostate cancer diagnosis and has significant risk of tumor recurrence and death compared with low/intermediate‐risk cancer.^1^, ^2^ Definitive treatment of high‐risk localized prostate cancer consists of radical prostatectomy (RP), external beam radiotherapy (EBRT) alone, or EBRT plus brachytherapy boost (EBRT + BT). Androgen deprivation therapy (ADT) is routinely combined with radiotherapy (RT).^3^, ^4^ Optimal management of high‐risk prostate cancer remains controversial. Recently, three observational studies with similar design and inclusion criteria found divergent results. In a multi‐institutional retrospective study, Kishan et al showed a better cancer‐specific survival (CSS) and overall survival (OS) associated with EBRT + BT compared with RP or EBRT alone.^5^ In a National Cancer Database (NCDB) study, Ennis et al found no OS difference between EBRT + BT and RP, whereas EBRT alone was inferior to RP.^6^ Conversely, Berg et al re‐analyzed the NCDB data with restriction to younger patients (age ≤ 65 years) and found RP was associated with a better OS, compared with EBRT + BT.^7^ Those controversial findings reflect limitations of study methodology, including retrospective study nature, inherent patient selection bias associated with surgery vs radiotherapy decisions (eg, advanced age and comorbidity favoring radiotherapy), and patient heterogeneity requiring further optimization from parameters not incorporated in the current risk stratification system.

Given the lack of prospective clinical trials, well‐designed observational studies are required to address the aforementioned limitations and guide treatment decisions. For this study, we utilized the Surveillance, Epidemiology, and End Results (SEER) database to explore the survival outcomes of high‐risk localized prostate cancer by surgery, EBRT alone, or EBRT + BT approaches. To minimize selection bias associated with surgery vs RT, we selected our target patients as those who had surgery or who were recommended to have surgery but received RT instead, assuming the same surgical selection criteria applies. To balance covariates and reduce bias in treatment effect estimation, we used inverse probability of treatment weighing (IPTW) utilizing the propensity score.

## 2 PATIENTS AND METHODS

### 2.1 Data source

The SEER database is an authoritative source of national cancer incidence and survival. It collects patient‐level data in 18 cancer registries across the United States and captures 28% of the US population, which represents all American trends. For this analysis, we used the SEER 21 research dataset of November 2018 and identified prostate adenocarcinoma using ICD‐O‐3 codes. Variables included (a) tumor characteristics, such as T stage, PSA value, Gleason score, and tumor grade; (b) definitive treatment information, such as surgery, EBRT, and EBRT + BT; and (c) patient characteristics, such as age, race, year of diagnosis, geographic locations, and marital status.

### 2.2 Study population

**Figure 1:**
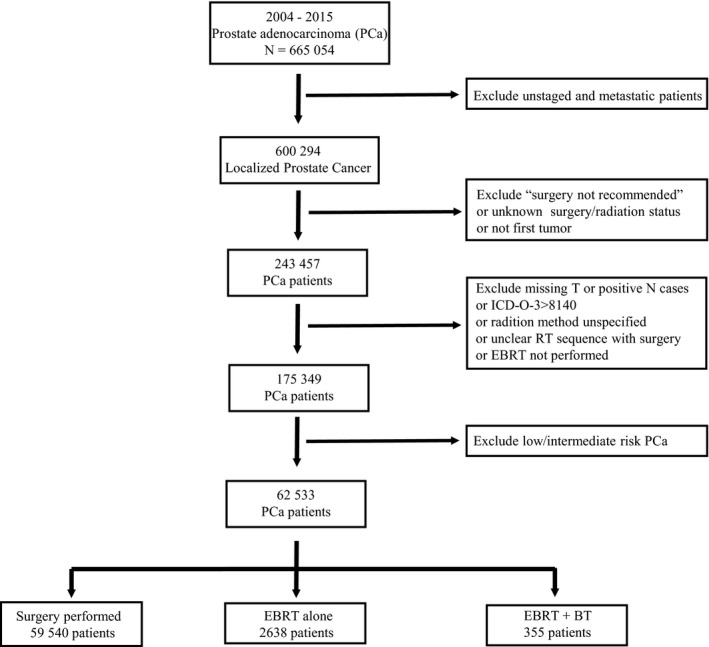
CONSORT diagram for the cohort analyzed

Figure 1 showed the method of patient selection. We included prostate adenocarcinoma patients with PSA > 20 ng/mL, or Gleason score ≥ 8, or clinical T stage ≥ 3, while patients with positive lymph nodes or distant metastasis were excluded. Although SEER database includes data dating back to the 1970s, we included patients diagnosed between 2004 and 2015 to provide contemporary treatment strategies and to be consistent with the three recent studies.^5^, ^6^, ^7^ The coding of surgery for primary site by SEER can be divided into five categories: (a) surgery was performed; (b) surgery was recommended but was not performed; (c) surgery was not recommended and was not performed; (d) it is unknown if surgery was performed; and (e) surgery was not performed, and patient died prior to recommended surgery. We included patients of category (a), and (b) if RT was performed. Notably, although SEER did not collect ADT treatment information, we assume that the majority of RT patients received concurrent ADT therapy since it became the standard of care in high‐risk prostate cancer by the time this patient population was treated.

### 2.3 Statistical analysis

#### 2.3.1 Data processing

Logistic regression was used to associate baseline covariates with treatment assignment. Cox proportional hazard regression was applied to find associations between baseline covariates including treatment groups and outcomes, which are OS and CSS. Covariates that were related to both treatment and outcome were included in the propensity score model. After risk stratification with crude data, we imputed missing values in variables of PSA and tumor grade (missing > 1% within groups) through Multivariate Imputation by Chained Equations for analysis purpose. Propensity scores were estimated with the generalized boosted model (GBM). Two stopping rules, mean of standardized mean differences (SMD) and max of Kolmogorov‐Smirnov (KS) statistics across covariates, were monitored to assess the balance of covariates. Inverse probability of treatment weights (IPTW) were calculated from the estimated propensity score of GBM.

#### 2.3.2 Survival analysis

Covariate adjusted survival curves were generated with Kaplan‐Meier methods with inverse probability of treatment weights. IPTW‐weighted Cox proportional hazard regression models were used to obtain hazard ratios (HRs) with 95% confidence intervals (CI).

#### 2.3.3 Meta‐analysis

We performed a meta‐analysis to estimate pooled HRs associated with CSS and OS. If the HR for related comparison is not provided in the original articles, we reconstructed time‐to‐event data from published Kaplan‐Meier curves.^8^ We assessed the between‐study heterogeneity by using the Cochran Q test with a significance level of *P* < .05. We performed initial analyses with a fixed‐effect model, and confirmatory analyses were performed with a random‐effect model if there was significant heterogeneity.

## 3 RESULTS

### 3.1 Patient characteristics

Clinical and pathological characteristics are shown in Table 1. A total of 62 533 patients were eligible for final analysis with a median age of 64 years old. The majority of patients were Caucasian (69.1%), followed by African American (23.7%) and other ethnicities (7.2%). Patients treated with surgery were significantly younger than those treated with RT, and were less likely to have a high PSA (>20 ng/mL) or Gleason score (>7) (*P* < .01 for all comparisons). African Americans and patients in the geographic locations of Southwest, Midwest and East were more likely to have RT, instead of recommended surgery (RT percentage: 8.5%, 17.2%, 9.6%, and 8.7%, respectively; *P* < .01 for all comparisons), while patients with partner support (married or domestic partner) were less likely to forgo surgery than those without partners (3.8% vs 7.6%, *P* < .01). About 59 540 patients had primary surgery with 8084 salvage RT treatment, while 2638 underwent EBRT alone and 355 underwent EBRT + BT. The median follow‐up durations were 58 months (interquartile range, 28‐92) for surgery; 62 months (interquartile range, 33‐60) for EBRT alone; and 87 months (interquartile range, 55‐117) for EBRT + BT. Table S1 showed the hazard ratios associated with selected clinicopathological factors.

**Table 1:** Characteristics of 62 533 high‐risk prostate cancer patients

	EBRT	EBRT + BT	Surgery
Patients, n	2638	355	59 540
Mean age (years)	69.4	66.1	63.8
Age group, n (%)			
<50	27 (1.0)	6 (1.7)	2038 (3.4)
50‐65	818 (31.0)	165 (46.5)	33 343 (56.0)
66‐75	1151 (43.6)	139 (39.2)	19 507 (32.8)
>75	642 (24.3)	45 (12.7)	4652 (7.8)
Ethnicity, n (%)			
White	1809 (68.6)	259 (73.0)	47 699 (80.1)
Black	638 (24.2)	71 (20.0)	7663 (12.9)
Other	133 (5.0)	22 (6.2)	3675 (6.2)
Unknown	58 (2.2)	3 (0.8)	503 (0.8)
Tumor grade, n (%)			
Grade I	19 (0.7)	1 (0.3)	466 (0.8)
Grade II	268 (10.2)	34 (9.6)	10 827 (18.2)
Grade III/IV	2351 (89.1)	320 (90.1)	48 247 (81.0)
T stage, n (%)			
T1	1287 (48.8)	162 (45.6)	3730 (6.3)
T2	1071 (40.6)	151 (42.5)	15 041 (25.3)
T3	259 (9.8)	41 (11.5)	37 210 (62.5)
T4	21 (0.8)	1 (0.3)	3559 (6.0)
Location, n (%)			
East	932 (35.3)	61 (17.2)	9353 (15.7)
West	675 (25.6)	93 (26.2)	29 070 (48.8)
Midwest	499 (18.9)	115 (32.4)	6466 (10.9)
South	214 (8.1)	60 (16.9)	12 098 (20.3)
Southwest	266 (10.1)	7 (2.0)	1316 (2.2)
Others	52 (2.0)	19 (5.4)	1237 (2.1)
PSA (ng/dl), n (%)			
<10	1166 (44.2)	177 (49.9)	38 312 (64.3)
10‐20	487 (18.5)	64 (18.0)	10 622 (17.8)
>20	985 (37.3)	114 (32.1)	10 606 (17.8)
Gleason score, n (%)			
≤6	173 (6.6)	27 (7.6)	7874 (13.2)
7	477 (18.1)	63 (17.7)	25 834 (43.4)
8‐10	1976 (74.9)	265 (74.6)	25 616 (43.0)
Unknown	12 (0.5)	0 (0.0)	216 (0.4)
Marital status, n (%)			
No partner	476 (18.0)	52 (14.6)	6374 (10.7)
With partner	1435 (54.4)	221 (62.3)	43 933 (73.8)
Single	297 (11.3)	28 (7.9)	5945 (10.0)
Unknown	430 (16.3)	54 (15.2)	3288 (5.5)

### 3.2 Survival outcomes by treatment strategies

**Figure 2:**
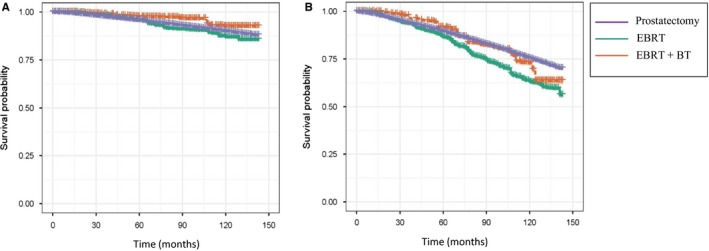
Inverse probability of treatment weighting‐adjusted Kaplan‐Meier curves stratified by the three treatments. A. cancer‐specific survival; B, overall survival

As shown in Table 2 and Figure 2, patients treated with EBRT + BT had significantly better cancer‐specific survival, compared with patients treated with surgery or EBRT alone (EBRT + BT vs surgery: HR, 0.55, 95% CI, 0.3‐1.00; EBRT + BT vs EBRT: HR, 0.49, 95% CI, 0.24‐0.98). No difference was found between EBRT and surgery by CSS outcome. When all‐cause mortality is considered, surgery had a significantly better OS than EBRT and a similar OS as EBRT + BT (EBRT vs surgery: HR, 1.46, 95% CI, 1.16‐1.8; EBRT + BT vs surgery: HR, 1.08, 95% CI, 0.68‐1.72). These results suggested substantially elevated competing causes of death other than prostate cancer in patients treated with radiation (EBRT or EBRT + BT) over time. We then examined if salvage RT impacted the surgery vs RT comparisons by removing salvage RT patients from the surgery group. The conclusions were not substantially changed, although the statistical significance was lost in comparison of cancer‐specific mortality between EBRT + BT and surgery (Table 2). Since patients who forwent prostatectomy for RT were more likely to be older and susceptible to other causes of death, we performed stratified analyses by age (≤65 years or >65 years). In patients ≤65 years, EBRT was significantly inferior to surgery in both cancer‐specific mortality and all‐cause mortality. In patients >65 years, EBRT was still inferior to surgery in all‐cause mortality, but EBRT and EBRT + BT showed a nonsignificant reduced cancer‐specific mortality compared with surgery. When combined, RT (EBRT and EBRT + BT) was statistically significantly associated with a better cancer‐specific mortality (HR, 0.69, 95% CI, 0.49‐0.97).

**Table 2:** Hazard ratios of cancer‐specific mortality and all‐cause mortal

	Cancer‐specific mortality	*P*	Overall mortality	*P*
	HR (95% CI)		HR (95% CI)	
All Patients				
EBRT vs Surgery	1.13 (0.8‐1.59)	.492	1.46 (1.16‐1.8)	<.001
EBRT + BT vs Surgery	0.55 (0.3‐1.00)	.051	1.08 (0.68‐1.72)	.749
EBRT + BT vs EBRT	0.49 (0.24‐0.98)	.043	0.74 (0.44‐1.24)	.251
Exclude Salvage RT				
EBRT vs Surgery	1.23 (0.87‐1.73)	.239	1.50 (1.22‐1.85)	<.001
EBRT + BT vs Surgery	0.6 (0.33‐1.09)	.095	1.11 (0.7‐1.77)	.662
Age ≤ 65 years				
EBRT vs Surgery	1.83 (1.04‐3.21)	.036	1.67 (1.11‐2.53)	.014
EBRT + BT vs Surgery	0.71 (0.28‐1.83)	.482	1.49 (0.71‐3.13)	.290
EBRT + BT vs EBRT	0.39 (0.13‐1.18)	.096	0.89 (0.38‐2.09)	.790
RT vs Surgery	1.57 (0.95‐2.61)	.080	1.63 (1.13‐2.34)	.008
Age > 65 years				
EBRT vs Surgery	0.73 (0.50‐1.05)	.085	1.23 (1.00‐1.5)	.046
EBRT + BT vs Surgery	0.49 (0.23‐1.03)	.058	0.91 (0.51‐1.6)	.733
EBRT + BT vs EBRT	0.67 (0.29‐1.54)	.347	0.74 (0.41‐1.35)	.325
RT vs Surgery	0.69 (0.49‐0.97)	.031	1.18 (0.97‐1.42)	.094

### 3.3 Meta‐analysis

Table 3 summarized the four available observational studies (including ours) in assessment of surgery vs EBRT ± BT for high‐risk prostate cancer treatment. The two studies utilizing CSS as the endpoint consistently showed a better outcome with EBRT + BT. All three studies showed an inferior outcome with EBRT. The two studies performed in younger patients (≤65 years) showed a better outcome with surgery by OS.

**Table 3:** Studies comparing surgery vs EBRT vs EBRT + BT in high‐risk prostate cancer

Study	Data source	Year of diagnosis	Age (years)	Patient no. (RP/EBRT/EBRT + BT)	Group of comparisons	Result (CSS)	Result (OS)
Berg, 2018	NCDB	2004‐2010	≤ 65	13 985 (12 283/0/1702)	EBRT + BT vs RP	NA	Favor RP
Kishan, 2018	Multi‐institutions	2000‐2013	All age	1809 (639/734/436)	EBRT + BT vs EBRT vs RP	Favor EBRT + BT	≤7.5 y, favor EBRT + BT >7.5 y, no difference
Ennis, 2018	NCDB	2004‐2013	All age	42 765 (24 688/15 435/2642)	EBRT + BT vs EBRT vs RP	NA	Equal EBRT + BT vs RP EBRT inferior
Ours, 2019	SEER	2004‐2015	All age	62 533 (59 540/2638/355)	EBRT + BT vs EBRT vs RP	favor EBRT + BT	EBRT inferior

Note

Comparison: EBRT vs Surgery; EBRT + BT vs Surgery; EBRT + BT vs EBRT.

**Figure 3:**
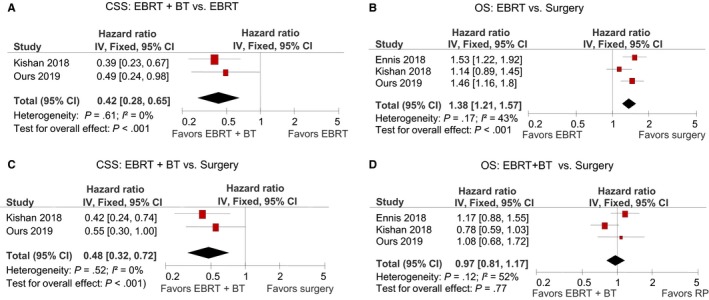
Meta‐analysis results. A. EBRT + BT vs EBRT by CSS; B, EBRT vs Surgery by OS; C, EBRT + BT vs Surgery by CSS; D. EBRT + BT vs Surgery by OS

To generate a higher level of evidence, we performed quantitative analyses of data from three observational studies (Ennis et al, Kishan et al, and ours). As shown in Table 4 and Figure 3, EBRT + BT was associated with a significantly reduced cancer‐specific mortality, compared with surgery or EBRT alone. There was no significant difference between surgery and EBRT alone by CSS outcome. However, surgery was associated with a significantly reduced all‐cause mortality, compared with EBRT alone. There was no significant difference between surgery and EBRT + BT by OS outcome.

**Table 4:**
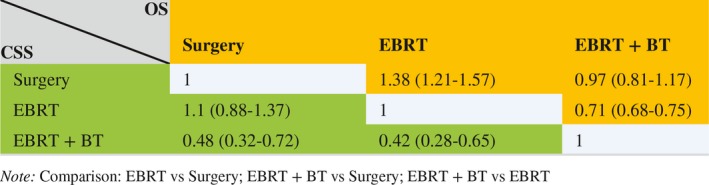
Meta‐analysis results

## 4 DISCUSSION

Analyses of large, population‐based cancer patient cohorts with good study design can provide important insights into real‐world treatment outcomes. The current study of 62 533 high‐risk localized prostate cancer patients from the SEER database aims to provide new evidence in comparing clinical outcomes of surgery, EBRT alone and EBRT + BT. Our study demonstrated that EBRT + BT was associated with a superior CSS compared with surgery or EBRT alone, whereas surgery and EBRT + BT had similar yet superior OS than EBRT. These results are congruent with the quantitative meta‐analysis of the three largest observational studies that utilized similar study design and methodology. Overall, treatment of EBRT + BT is associated with significantly better outcomes in the high‐risk patient population.

Patient selection bias is one of the major limitations of retrospective cohort studies.^9^ Many observational studies utilized propensity score to balance covariates, but they are limited in the number of collected variables to inform treatment decisions. Some parameters (eg, ECOG performance status and Charlson Comorbidity Index) have been used to control general health status, but they are not designed to determine the surgical eligibility and their prognostic relevance for surgery is restricted to patients older than 70 years.^10^ Thus, there can still be significant difference in patient characteristics between surgery and RT patients. Our study has been designed to reduce selection bias by (a) focusing on surgical candidates to ensure a uniform patient population based on surgery selection criteria, (b) omitting patients with positive clinical lymph nodes because RT is usually preferred in such patients, (c) utilizing analysis method of inverse probability of treatment weighing. Balance was assessed using standardized differences, which revealed <15% imbalance for all covariates (majority <10%) between surgery and RT patients. Additionally, we performed a meta‐analysis to combine studies utilizing similar design (surgery vs EBRT + BT vs EBRT) and methodology (IPTW‐weighted Cox proportional hazard regression models) to inform a higher level of evidence. Those procedures help to boost the relevance and reliability of our findings.

In this study, we found a better CSS to be associated with EBRT + BT, compared with EBRT or surgery. However, the survival benefit was lost when all‐cause mortality was considered. This observation suggested either a long‐term harmful effects of radiotherapy, or the existence of other harmful factors associated with radiotherapy (eg, ADT treatment) contributing to increased noncancer‐related mortality, because the death risks elevated from CSS to OS in both EBRT + BT and EBRT groups, when compared with surgery. Indeed, multiple treatment‐related complications can negatively impact life expectancy of prostate cancer patients treated with RT + ADT, including RT‐induced second malignancies and an array of significant debilitating adverse effects by ADT, such as body composition changes, psychological and cognitive defects, metabolic disturbances, and cardiovascular morbidity.^11^, ^12^ It is also possible that other age‐related cause of mortality rose significantly over time, which abrogated the cancer‐specific mortality benefit derived from EBRT + BT, since patients with RT therapy were older. A significantly increased all‐cause mortality was seen with EBRT + BT compared to surgery in patients ≤65 years;^7^ hence, we tested this hypothesis by dividing patients into two groups based on age (≤ 65 or >65 years). EBRT was inferior to surgery in both age groups. There was a trend for increased all‐cause mortality in EBRT + BT compared to surgery in patients ≤65, but the study power was limited to detect the difference. Interestingly, both EBRT and EBRT + BT favored a better CSS in patients >65 years compared to surgery, which was statistically significant when the two groups were combined. Considering that more patients treated with RT had PSA >20 ng/mL or Gleason score > 7, these results are compelling to favor RT. Additional studies are required to understand the mechanisms and determine if RT should be preferred in this age group. Separately, the meta‐analysis seemed to support EBRT + BT>RP > EBRT in clinical outcomes of all‐age patients. Overall, our results may provide clues to reconcile the differences between the previous three observational studies,^13^, ^14^ yet pose new findings and questions for the optimal management of high‐risk localized prostate cancer.

Our study has some limitations. First, our study is neither prospective nor randomized. However, real‐world data mirror the outcomes of daily practice, and its study helps leverage available evidence to inform treatment decisions and to provide valuable information to guide future trial designs. As examples, EBRT + BT should not be assumed to be equivalent to EBRT, and should be designed as independent arms. Patient's age could be key in determining treatment strategy, because of the possibility of competing causes of death in older individuals or the better CSS seen in patients over 65 years treated with radiotherapy. Second, some important information is not available because the SEER database does not collect data such as RT dosage, ADT treatment, type of surgery, and treatment toxicities. However, our conclusions are not likely to change after adjustment of those factors because the impact of inadequate surgery (likely a small number) will be diluted to the minimum given the large number of patients in surgery group and inadequate ADT treatment will only compromise efficacy of radiation. Third, although some prior studies compared the effectiveness of surgery vs RT in localized prostate cancer,^15^, ^16^, ^17^, ^18^, ^19^, ^20^ we decided to exclude them from our meta‐analysis because (a) a few studies were performed in high‐risk prostate cancer and even fewer assessed EBRT + BT as an independent treatment group, (b) many study patients were diagnosed in the 1980s or 1990s, which may not be comparable to the patient population of the three observational studies (diagnosed after 2000), and (C) none used IPTW‐weighed regression models. Hence, our meta‐analysis only included a limited number of studies. Fourth, we acknowledge that the majority of patients included in this study had surgery, which outnumbered the patients undergoing RP. This is due to our study design of targeting surgical candidates as the study population. Hence, we utilized IPTW‐weighted regression model to reduce the imbalance between surgery and RP groups.

In conclusion, our study showed that EBRT + BT was associated with significantly better prostate cancer‐specific survival and similar overall survival compared with surgery. EBRT alone was inferior to surgery by overall survival. Prospective clinical trials should be designed to better understand the optimal treatment approach.

## CONFLICT OF INTEREST

Monika Joshi is a consultant of Bayer and Sanofi, and have research grants sponsored by Pfizer and Astrazeneca. Amir Mortazavi serves in advisory board of Seattle Genetics, Janssen, Debiopharm Group. All others have no conflict of interest relevant to the subject matter or materials discussed in the manuscript.

## AUTHOR CONTRIBUTIONS

Ming Yin and Steven Clinton conceptualized the study. Ming Yin collected the data and involved in writing original draft. Ming Yin and Jing Zhao performed statistical analysis. Jing Zhao, Paul Monk, Douglas Martin, Edmund Folefac, Monika Joshi, Ning Jin, Amir Mortazavi, Clair Verschraegen, and Steven Clinton wrote, reviewed, and edited the manuscript.

## Data Availability

The data that support the findings of this study are openly available in the SEER database.

## References

[1] Cooperberg MR , Broering JM , Carroll PR . Time trends and local variation in primary treatment of localized prostate cancer. J Clin Oncol. 2010;28(7):1117‐1123. 20124165 10.1200/JCO.2009.26.0133 PMC2834465

[2] Cooperberg MR , Vickers AJ , Broering JM , Carroll PR . Comparative risk‐adjusted mortality outcomes after primary surgery, radiotherapy, or androgen‐deprivation therapy for localized prostate cancer. Cancer. 2010;116(22):5226‐5234. 20690197 10.1002/cncr.25456 PMC2975879

[3] Lawton C , Lin X , Hanks GE , et al. Duration of androgen deprivation in locally advanced prostate cancer: long‐term update of NRG oncology RTOG 9202. Int J Radiat Oncol Biol Phys. 2017;98(2):296‐303. 28463149 10.1016/j.ijrobp.2017.02.004 PMC5603177

[4] Zapatero A , Guerrero A , Maldonado X , et al. High‐dose radiotherapy with short‐term or long‐term androgen deprivation in localised prostate cancer (DART01/05 GICOR): a randomised, controlled, phase 3 trial. Lancet Oncol. 2015;16(3):320‐327. 25702876 10.1016/S1470-2045(15)70045-8

[5] Kishan AU , Cook RR , Ciezki JP , et al. Radical prostatectomy, external beam radiotherapy, or external beam radiotherapy with brachytherapy boost and disease progression and mortality in patients with gleason score 9–10 prostate cancer. JAMA. 2018;319(9):896‐905. 29509865 10.1001/jama.2018.0587 PMC5885899

[6] Ennis RD , Hu L , Ryemon SN , Lin J , Mazumdar M . Brachytherapy‐based radiotherapy and radical prostatectomy are associated with similar survival in high‐risk localized prostate cancer. J Clin Oncol. 2018;36(12):1192‐1198. 29489433 10.1200/JCO.2017.75.9134

[7] Berg S , Cole AP , Krimphove MJ , et al. Comparative effectiveness of radical prostatectomy versus external beam radiation therapy plus brachytherapy in patients with high‐risk localized prostate cancer. Eur Urol. 2019;75(4):552‐555. 30420255 10.1016/j.eururo.2018.10.032

[8] Wei Y , Royston P . Reconstructing time‐to‐event data from published Kaplan‐Meier curves. Stata J. 2017;17(4):786‐802. 29398980 PMC5796634

[9] Soni PD . Selection bias in population registry‐based comparative effectiveness research. Int J Radiat Oncol Biol Phys. 2019;103(5):1058‐1060. 30900558 10.1016/j.ijrobp.2018.12.011

[10] Froehner M , Koch R , Litz RJ , Oehlschlaeger S , Hakenberg OW , Wirth MP . Feasibility and limitations of comorbidity measurement in patients undergoing radical prostatectomy. Eur Urol. 2005;47(2):190‐195. 15661413 10.1016/j.eururo.2004.07.031

[11] Sountoulides P , Rountos T . Adverse effects of androgen deprivation therapy for prostate cancer: prevention and management. ISRN Urol. 2013;2013:240108. 23984103 10.1155/2013/240108 PMC3747499

[12] Sountoulides P , Koletsas N , Kikidakis D , Paschalidis K , Sofikitis N . Secondary malignancies following radiotherapy for prostate cancer. Ther Adv Urol. 2010;2(3):119‐125. 21789089 10.1177/1756287210374462 PMC3126090

[13] Kishan AU , Cook RR , King CR . Reply to Marieke J. Krimphove, Junaid Nabi, Alexander P. Cole, and Quoc‐Dien Trinh's Letter to the Editor re: Ronald D. Ennis, Liangyuan Hu, Shannon N. Ryemon, Joyce Lin, Madhu Mazumdar. Brachytherapy‐based radiotherapy and radical prostatectomy are associated with similar survival in high‐risk localized prostate cancer. J Clin Oncol 2018;36:1192–8. Eur Urol Oncol. 2019;2(2):224‐225. 31017101 10.1016/j.euo.2018.08.019

[14] Ennis RD , Hu L , Ryemon SN , Lin J , Mazumdar M . Reply to Marieke J. Krimphove, Junaid Nabi, Alexander P. Cole, and Quoc‐Dien Trinh's Letter to the Editor re: Ronald D. Ennis, Liangyuan Hu, Shannon N. Ryemon, Joyce Lin, Madhu Mazumdar. Brachytherapy‐based radiotherapy and radical prostatectomy are associated with similar survival in high‐risk localized prostate cancer. J Clin Oncol 2018;36:1192–8. Eur Urol Oncol. 2019;2(2):226‐227. 31017102 10.1016/j.euo.2018.08.025

[15] Westover K , Chen M‐H , Moul J , et al. Radical prostatectomy vs radiation therapy and androgen‐suppression therapy in high‐risk prostate cancer. BJU Int. 2012;110(8):1116‐1121. 22540922 10.1111/j.1464-410X.2012.11012.x

[16] Kibel AS , Ciezki JP , Klein EA , et al. Survival among men with clinically localized prostate cancer treated with radical prostatectomy or radiation therapy in the prostate specific antigen era. J Urol. 2012;187(4):1259‐1265. 22335870 10.1016/j.juro.2011.11.084

[17] Lee JY , Cho KS , Kwon JK , et al. A competing risk analysis of cancer‐specific mortality of initial treatment with radical prostatectomy versus radiation therapy in clinically localized high‐risk prostate cancer. Ann Surg Oncol. 2014;21(12):4026‐4033. 24841351 10.1245/s10434-014-3780-9

[18] Boorjian SA , Karnes RJ , Viterbo R , et al. Long‐term survival after radical prostatectomy versus external‐beam radiotherapy for patients with high‐risk prostate cancer. Cancer. 2011;117(13):2883‐2891. 21692049 10.1002/cncr.25900 PMC3139725

[19] Akakura K , Suzuki H , Ichikawa T , et al. A randomized trial comparing radical prostatectomy plus endocrine therapy versus external beam radiotherapy plus endocrine therapy for locally advanced prostate cancer: results at median follow‐up of 102 months. Jpn J Clin Oncol. 2006;36(12):789‐793. 17082219 10.1093/jjco/hyl115

[20] Merglen A , Schmidlin F , Fioretta G , et al. Short‐ and long‐term mortality with localized prostate cancer. Arch Intern Med. 2007;167(18):1944‐1950. 17923593 10.1001/archinte.167.18.1944

