# Supplementary material for: Comparative effectiveness of surgery versus external beam radiation with/without brachytherapy in high‐risk localized prostate cancer
Source: Cancer Med. 2019 Nov 7;9(1):27–34. doi: 10.1002/cam4.2605 (PMC6943084; doi:10.1002/cam4.2605)
Supplement: Supplementary file 1 [file CAM4-9-27-s001.docx]

| **Supplementary Table 1** Hazard ratios of cancer-specific mortality and all-cause mortality with selected clinicopathologial factors | | | | |
| --- | --- | --- | --- | --- |
| **Cancer-specific mortality** | | | **Overall mortality** | |
|  | **HR (95% CI)** | ***P*** | **HR (95% CI)** | ***P*** |
| Age |  |  |  |  |
| < 50 | 1 |  |  |  |
| 50 – 65 | 1.13 (0.87-1.46) | 0.357 | 1.50 (1.25-1.80) | <0.001 |
| 66 – 75 | 1.62 (1.25-2.11) | <0.001 | 2.82 (2.34-3.39) | <0.001 |
| > 75 | 6.13 (4.71-7.98) | <0.001 | 10.44 (8.66-12.60) | <0.001 |
| PSA (ng/dl) |  |  |  |  |
| <10 | 1 |  | 1 |  |
| 10 – 20 | 1.34 (1.21-1.48) | <0.001 | 1.32 (1.25-1.41) | <0.001 |
| > 20 | 2.35 (2.16-2.56) | <0.001 | 1.97 (1.86-2.07) | <0.001 |
| Gleason score |  |  |  |  |
| ≤ 6 | 1 |  | 1 |  |
| 7 | 1.34 (1.03-1.75) | 0.030 | 1.22 (0.99-1.26) | 0.058 |
| 8-10 | 4.96 (3.82-6.43) | <0.001 | 2.17 (1.92-2.44) | <0.001 |
| T stage |  |  |  |  |
| T1-T3 | 1 |  | 1 |  |
| T4 | 2.66 (2.35-3.00) | <0.001 | 1.77 (1.62-1.94) | <0.001 |
| Tumor Grade |  |  |  |  |
| Grade I | 1 |  | 1 |  |
| Grade II | 0.88 (0.41-1.92) | 0.750 | 0.75 (0.53-1.07) | 0.115 |
| Grade III/IV | 1.55 (0.72-3.32) | 0.262 | 0.89 (0.63-1.27) | 0.526 |
